# Clinical features and genetic background of the sympatric species *Paracoccidioides brasiliensis* and *Paracoccidioides americana*

**DOI:** 10.1371/journal.pntd.0007309

**Published:** 2019-04-15

**Authors:** Priscila Marques de Macedo, Marcus de Melo Teixeira, Bridget M. Barker, Rosely Maria Zancopé-Oliveira, Rodrigo Almeida-Paes, Antonio Carlos Francesconi do Valle

**Affiliations:** 1 Infectious Dermatology Clinical Research Laboratory, Evandro Chagas National Institute of Infectious Diseases, Fiocruz, Rio de Janeiro, Rio de Janeiro, Brazil; 2 Faculty of Medicine, University of Brasília, Brasília, Federal District, Brazil; 3 Pathogen and Microbiome Institute, Northern Arizona University, Flagstaff, Arizona, United States of America; 4 Mycology Laboratory, Evandro Chagas National Institute of Infectious Diseases, Fiocruz, Rio de Janeiro, Rio de Janeiro, Brazil; Faculty of Science, Ain Shams University (ASU), EGYPT

## Abstract

**Introduction:**

The agents of paracoccidioidomycosis, historically identified as *Paracoccidioides brasiliensis*, are in fact different phylogenetic species. This study aims to evaluate associations between *Paracoccidioides* phylogenetic species and corresponding clinical data.

**Methods:**

*Paracoccidioides* strains from INI/Fiocruz patients (1998–2016) were recovered. Socio-demographic, epidemiological, clinical, serological, therapeutic and prognostic data of the patients were collected to evaluate possible associations of these variables with the fungal species identified through partial sequencing of the ADP-ribosylation factor (*arf*) and the 43-kDa-glycoprotein (*gp43*) genes.

**Results:**

Fifty-four fungal strains were recovered from 47 patients, most (72.3%) infected in Rio de Janeiro state, Brazil. Forty-one cases were caused by *Paracoccidioides brasiliensis* and six by *Paracoccidioides americana* (former PS2). *P*. *brasiliensis* was responsible for severe lymph abdominal forms, whereas patients infected with *P*. *americana* presented a high rate of adrenal involvement. However, no statistically significant associations were found for all variables studied. *P*. *americana* presented 100% reactivity to immunodiffusion, even when tested against antigens from other species, while negative results were observed in 9 (20%) cases caused by *P*. *brasiliensis*, despite being tested against a homologous antigen.

**Conclusions:**

*P*. *brasiliensis* and *P*. *americana* are sympatric and share similar clinical features and habitat, where they may compete for similar hosts.

## Author summary

Paracoccidioidomycosis (PCM) is a severe systemic mycosis caused by different phylogenetic species. According to previous studies, these species could have an impact in PCM clinical features. This study aims to investigate possible associations between *Paracoccidioides* species and corresponding clinical data. The fungal strains from the patients were recovered, whereas their clinical data were collected to evaluate possible associations of these variables with the fungal species identified through DNA sequencing. Fifty-four fungal strains were recovered from 47 patients, most infected in Rio de Janeiro state, Brazil. Forty-one cases were caused by *Paracoccidioides brasiliensis* and six by *Paracoccidioides americana*. *P*. *brasiliensis* was responsible for severe clinical forms, and patients infected with *P*. *americana* presented a high rate of adrenal involvement. However, no statistically significant associations were found for all variables studied. *P*. *brasiliensis* and *P*. *americana* share similar clinical features.

## Introduction

Paracoccidioidomycosis (PCM) is a mycotic disease with a large spectrum of clinical presentations that affects both immunocompetent and immunocompromised patients from different biomes of Latin America [1]. The infection primarily affects the lungs and is acquired by the inhalation of *Paracoccidioides* sp. conidia or mycelial fragments that becomes aerosolized after the soil perturbation [2, 3]. Once inside the host, the fungus differentiates into pathogenic multi-budding yeast-like cells and this dimorphic process may lead to pathogenesis [4]. Incidence in endemic areas is associated with deforestation, armadillo hunting, and agriculture practices [1]. Massive soil removal during construction was recently suggested as another risk factor for the acquisition of PCM when a localized epidemic was recently reported among people living near highway construction [5]. Natural infections in a wide variety of animals have been reported in the literature, but recurrent isolation of the fungus from armadillos (*Xenarthra* superorder.) has led many authors to suggest that these mammals play an important role in the fungal life cycle and dispersion [6].

The infection can be controlled and/or cleared by the innate immune system after inhalation, and is thought to be the main reason why the majority of infections are asymptomatic [7]. However, it may progress to an acute/sub-acute disseminated pathology that may affect lymph nodes, liver, spleen, gut, bones joints, and meninges or to a chronic pulmonary disease [7]. The acute/sub-acute form may have several complications and sequelae such as low adrenal reserve, lymphedema, spleen calcifications, among others [8]. Moreover, the chronic pulmonary form of the disease can be disabling due to pulmonary fibrosis development, and is frequently seen in PCM endemic areas of Brazil, Argentina, Venezuela, and Colombia that report the majority of cases [9]. The number of early deaths, even in patients with unknown previous clinical history, is remarkably high and this mycotic disease is the most common cause of hospitalization due to fungal infections in immunocompetent patients in Brazil [10]. The severity of specific cases, the broad spectrum of clinical manifestations, and the highly variable immune response observed in patients with PCM requires further investigation of the organism’s genetic contribution to disease plasticity, diagnostics, and prognostics [11].

The etiological agents of human PCM are distributed into at least 5 species: *Paracoccidioides brasiliensis* (former S1 phylogenetic group), *Paracoccidioides americana* (former PS2 phylogenetic group), *Paracoccidioides restrepiensis* (former PS3 phylogenetic group), *Paracoccidioides venezuelensis* (former PS4 phylogenetic group), and *Paracoccidioides lutzii* (former *Pb*01-like phylogenetic group) [11–17]. The species *P*. *restrepiensis* and *P*. *venezuelensis* are geographically restricted to Colombia and Venezuela respectively, while *P*. *brasiliensis*, *P*. *americana*, and *P*. *lutzii* have a broad occurrence in Latin American countries [14, 17]. Moreover, *P*. *brasiliensis* is composed of two cryptic populations: S1a and S1b, that are differentially prevalent along eastern Brazil and southern South America respectively [16, 17]. *P*. *americana* has been identified in eastern Brazil and in a single occurrence in Venezuela, but with a lower incidence compared to *P*. *brasiliensis* [12, 14, 15]. *P*. *lutzii* constitutes a single genotype that is endemic to the north-mid western part of Brazil and Ecuador and it is genetically distant from the former *P*. *brasiliensis* species complex that includes the above mentioned species [11, 13, 15].

To date, fewer than 200 *Paracoccidioides* spp. strains have being properly genotyped and the availability of data from specific endemic areas of South America are scarce [12, 13, 14]. Regional efforts to understand the genetic epidemiology of these pathogens are needed as disease variations among patients are evident, treatment outcome may differ for different fungal species, and there may be differences in the arsenal of virulence factors expressed during infections within and between species.

Approximately 80% of PCM cases are reported in the Brazilian territory [1, 10]. The Southeast region of Brazil includes the states of São Paulo, Rio de Janeiro, Espírito Santo, and Minas Gerais, which are historically important areas of high endemicity of the disease [1]. The state of Rio de Janeiro has the third highest number of hospitalizations due to PCM in Brazil [10]. The disease was first reported by Adolpho Lutz in 1908, and reporting over almost a century in this state reveals a strong association with rural lifestyle and farming [18, 19]. The southern Paraíba Valley and the Resende basin are areas of sugar cane, coffee plantations and deforestation for both agriculture and livestock production, and are related to the disease burden in this state [20]. Acute PCM cases are also frequently reported in Rio de Janeiro suggesting active and constant dispersion of the fungus among the population [5, 8]. The disease is highly endemic in the metropolitan area of the Rio de Janeiro state (municipalities of Rio de Janeiro, Duque de Caxias, Itaguaí, Magé, Cachoeiras de Macacu, Nova Iguaçu) as well as in the Paraiba Valley (Volta Redonda and Barra Mansa) [5, 8, 10]. However, hospitalizations due to PCM are recorded in the entire range of the state, even in mountain regions (Petrópolis and Teresópolis), mid-South (Vassouras and Paraíba do Sul) or North of the state (Campos dos Goytacazes and São Fidélis) suggesting that the fungus is endemic to the whole state or perhaps the patients may have migrated from the place of infection to other regions of the state. Currently, 14 strains from patients living at Rio de Janeiro have been genotyped and the clinical information reported, resulting in 13 identified as *P*. *brasiliensis* and one as *P*. *americana* [8, 21–25]. These species are potentially sympatric since they seem to inhabit the same geographical area, i.e. Rio de Janeiro and São Paulo states [8, 14, 22].

In order to investigate the genetic background of *Paracoccidioides* spp. and its possible medical associations in the endemic area of Rio de Janeiro, 54 clinical strains recovered from 47 patients presenting both chronic and acute forms of PCM were identified by molecular techniques and the epidemiologic, clinical, therapeutic, and serological features of the patients were associated with the identified species. We also compared the *P*. *brasiliensis P*. *americana* isolation ratio between São Paulo and Rio de Janeiro in order to better understand the ecology of those species complexes.

## Methods

### Fungal strains

Isolation of fungi was carried out from 1998 to 2016 from patients admitted at the Evandro Chagas National Institute of Infectious Diseases (INI/Fiocruz) for diagnosis and clinical management of PCM. *Paracoccidioides* strains were isolated for diagnostic purposes from different human clinical samples: oral or nasal mucosa, lymph nodes, sputum, bronchoalveolar lavage, skin lesions, and spleen. The strains were maintained under mineral oil and recovered for molecular analyses after subcultures in Potato Dextrose Agar incubated at 25°C for 30 days. The *Paracoccidioides* spp. colonies were then subcultured in Fava-Netto agar plates incubated at 37°C for 14 days and single colonies were collected to further characterize the *Paracoccidioides* species by molecular biology.

### Ethical statements

The use of anonymous patients’ data of the patients herein included was approved by the Ethics Committee Board of INI/Fiocruz (number CAAE: 42590515.0.0000.5262).

### Patients

All patients for whom at least one viable colony of *Paracoccidioides* sp. was cultured were included in this study. Medical records of the included patients were anonymously reviewed. Socio-demographic and epidemiologic data included age, sex, place of birth and residence of the patients, as well as the probable region where they became infected. The latter was inferred from the information provided by the patient such as place of birth, residence, development of risk activities such as agricultural and/or construction activities, or armadillo hunting. Clinical information included the PCM clinical form, the main organs affected, and the grade of disease’s severity according to Mendes et al. [26]. All patients underwent a standard routine clinical evaluation including physical examinations, blood tests [hematology, liver and renal function tests, Ouchterlony double immunodiffusion (ID) test for PCM, enzyme immunoassay tests for screening of HIV antibodies], parasitological stool analysis, acid-fast bacilli and culture of clinical specimens, chest radiography, and other imaging examinations when indicated [brain computerized tomography (CT), abdominal CT or ultrasonography]. The adrenal function was evaluated using the adrenocorticotropic hormone/ACTH stimulation test. Low adrenal reserve was defined as a cortisol normal basal level and levels lower than 20 mg/dl after 30 and 60 minutes of stimulation. Some of the above mentioned tests may not have been performed depending on test availability and patient’s consent. Therapeutic regimen was based on the Brazilian PCM guidelines [7] that were recently revised. The analyzed data included the drugs prescribed and the total time of treatment. Prognostic information was related to the patient’s outcome such as cure, relapses, complications, and death. Cure criteria considered clinical, radiological, and serological aspects [7].

### Serologic tests

Double-immunodiffusion (ID) assay was applied for specific antibody detection using a pool of crude antigens obtained from isolates Pb01 (*P*. *lutzii*) and Pb339 (*P*. *brasiliensis*). The sera were obtained from the patients followed: at admission (before treatment), every 3 months until cure was achieved, and every 6 months until patients’ discharge. A quantitative ID was performed, through 2-fold dilutions of sera in phosphate buffered saline solution. For comparison purposes, the serum titer of the first and last stored serum samples of each patient were compared.

### *Paracoccidioides* genotyping

The genomic DNA of each strain was extracted from yeast-like cultures according to Ferrer et al. [27] and quantified using the NanoVue Plus Spectrophotometer. Each DNA sample was used as template for the PCR amplifications of the partial ADP-ribosylation factor (*arf*) and the 43-kDa-glycoprotein (*gp43*) genes using the Platinum Taq DNA polymerase 2X PCR Master Mix. The mixture contained 5 μl of 10X reaction buffer solution, 1 μl of the forward (ARF-F 5’TCTCATGGTTGGCCTCGATGCTGCC3’ and gp43-E2F 5’CCAGGAGGCGTGCAGGTGTCCC3’) and reverse (ARF-R 5’GAGCCTCGACGACACGGTCACGATC3’ and gp43-E2R 5’GCCCCCTCCGTCTTCCATGTCC3’) primers (10 pM) as described elsewhere [21], 5 μl of deoxynucleoside triphosphate solution (0.2 mM), 2 μl of magnesium chloride solution (2 mM), 0.5 μl of *Taq* DNA polymerase (2.5 U), 100 ng of fungal genomic DNA, and ultrapure water in a final reaction volume of 50 μl. Annealing primer conditions and cycling were adapted from Teixeira and collaborators, as previously described [11, 21].

The nucleotide sequences were determined via automatic capillary Sanger sequencing in an ABI 3730*xl*- Applied Biosystems machine using the BigDye Terminator v3.1 cycle sequencing kit (Thermo Fisher Scientific, USA). Sequencing was performed using both forward and reverse primers [21] and nucleotide quality control was checked using Phred [28]; only called bases with a Phred score > 30 were considered for subsequent analysis. Representative sequences of *arf* and *gp43* loci, covering *P*. *brasiliensis* (S1a and S1b), *P*. *americana*, *P*. *restrepiensis*, *P*. *venezuelensis*, and *P*. *lutzii* were added to the dataset [11–13, 29] (S1 Table). The sequences were aligned using the ClustalW algorithm [30] implemented in the BioEdit software [31] and were manually inspected.

In order to genetically classify the *Paracoccidioides* sp. strains from Rio de Janeiro state, Maximum Likelihood (ML) methods were applied. Phylogenetic trees were calculated using the IQ-TREE software [32] and nucleotide substitution models were selected using ModelFinder [33]. Each isolate was assigned to each species/genotype and branch support was inferred using both ultrafast bootstraps [34] and Shimodaira–Hasegawa approximate likelihood ratio test (SH-aLRT). Trees as well branch supports were visualized using FigTree v1.4. The haplotype networks were produced to visualize the microevolution of both *P*. *brasiliensis* and *P*. *americana*. The distribution and diversity of haplotypes for the *arf* + *gp43* dataset was estimated using the software DnaSP, v 5 [35] and the Median-joining network was built and visualized used in Network, v 4, software (Fluxus Technology, Clare, Suffolk, England).

### Mapping *P*. *brasiliensis* and *P*. *americana* in southeastern Brazil

Geographical locations and the genotypic profile of human, environmental and armadillo strains as well soil and biopsies amplicons recovered from *P*. *americana and P*. *brasiliensis* samples from Southeast Brazil (Rio de Janeiro, São Paulo and Minas Gerais) were retrieved for counts [12, 14, 36–42]. These three states are located in the Southeastern part of Brazil, a hot spot of PCM, and contain the majority of currently genotyped strains of *Paracoccidioides*.

### Statistical analyses

The sociodemographic, epidemiological, clinical, and prognostic data of the included patients were represented as frequencies and their respective 95% confidence intervals. If the mean values of a variable in two independent groups had confidence intervals that do not overlap, then the difference between the groups was considered significant. Additionally, Fisher exact test was used in the comparison of categorical data, Student’s t test in the comparison of treatment times, and the Wilcoxon test in the comparison of anti-*Paracoccidioides* antibody serum titers before and after treatment. The proportions of both *P*. *brasiliensis* and *P*. *americana* in each sample state of Brazil were compared using N-1 Pearson’s Chi-Square test with a 95% confidence interval [43]. *P*. *brasiliensis* and *P*. *americana* distributions were tested for deviation from the hypothetical ratio of 1:1 using a chi-square test in the Microsoft Excel platform. Finally, we tested deviation from the 1:1 ratio of the overall counts for humans and armadillo for host-specificity tests. The environmental records were excluded from the statistical analyses due to low sample size. In all analyses, a *p*<0.05 was considered to be statistically significant.

## Results and discussion

### *Paracoccidioides* spp. recovery

Laboratorial storage of *Paracoccidioides* remains a challenge, and the recovery rate of the fungus is usually low. A previous study has reported a 26% recovery rate in a collection of 70 *P*. *brasiliensis* strains maintained under mineral oil for long periods of time. Moreover, only strains stored less than 10 years were viable [44]. In the current study, from 128 clinical *Paracoccidioides* spp. strains stored under mineral oil in the mycological culture collection of INI/Fiocruz, 54 (42%) remained viable after 1 to 17 years of storage and were recovered for molecular analysis, corresponding to a total of 47 patients.

### Molecular aspects and genetic distribution of *Paracoccidioides* spp. in Rio de Janeiro, Brazil

Phylogenetic analysis of 54 *Paracoccidioides* spp. clinical strains from Rio de Janeiro, Brazil indicates *P*. *brasiliensis* (n = 48) and *P*. *americana* (n = 6) as the causative agents of PCM in patients living in this state. Teixeira and collaborators [11] established that the partial sequencing of the genes *arf* and *gp43* is able to differentiate *P*. *lutzii* from the three *P*. *brasiliensis* phylogenetic species (S1, PS2, and PS3). Years later, when these phylogenetic species were elevated to the formal taxonomic species *P*. *brasiliensis*, *P*. *americana*, and *P*. *restrepiensis*, respectively, it was shown that phylograms using nuclear concatenated coding loci, including *arf* and *gp43*, can differentiate the newly described species [17]. In a clinical laboratory scenario, sequencing of several genes is a difficult task, and then the use of the two genes described in this work can facilitate *Paracoccidioides* species differentiation in a clinical setting. It is important to note that both *arf* and *gp43* genes show a negative value for Tagima’s D, indicating an excess of low polymorphisms relative to expectation [17]. Moreover, our phylogenetic identification is supported by phylogenetic and haplotype network analyses that included *arf* and *gp43* sequences derived from strains successfully identified in previous publications [11, 12, 17], which support the concept that the sequencing of these two genes allows the differentiation between *P*. *brasiliensis* and *P*. *americana*.

**Fig 1:**
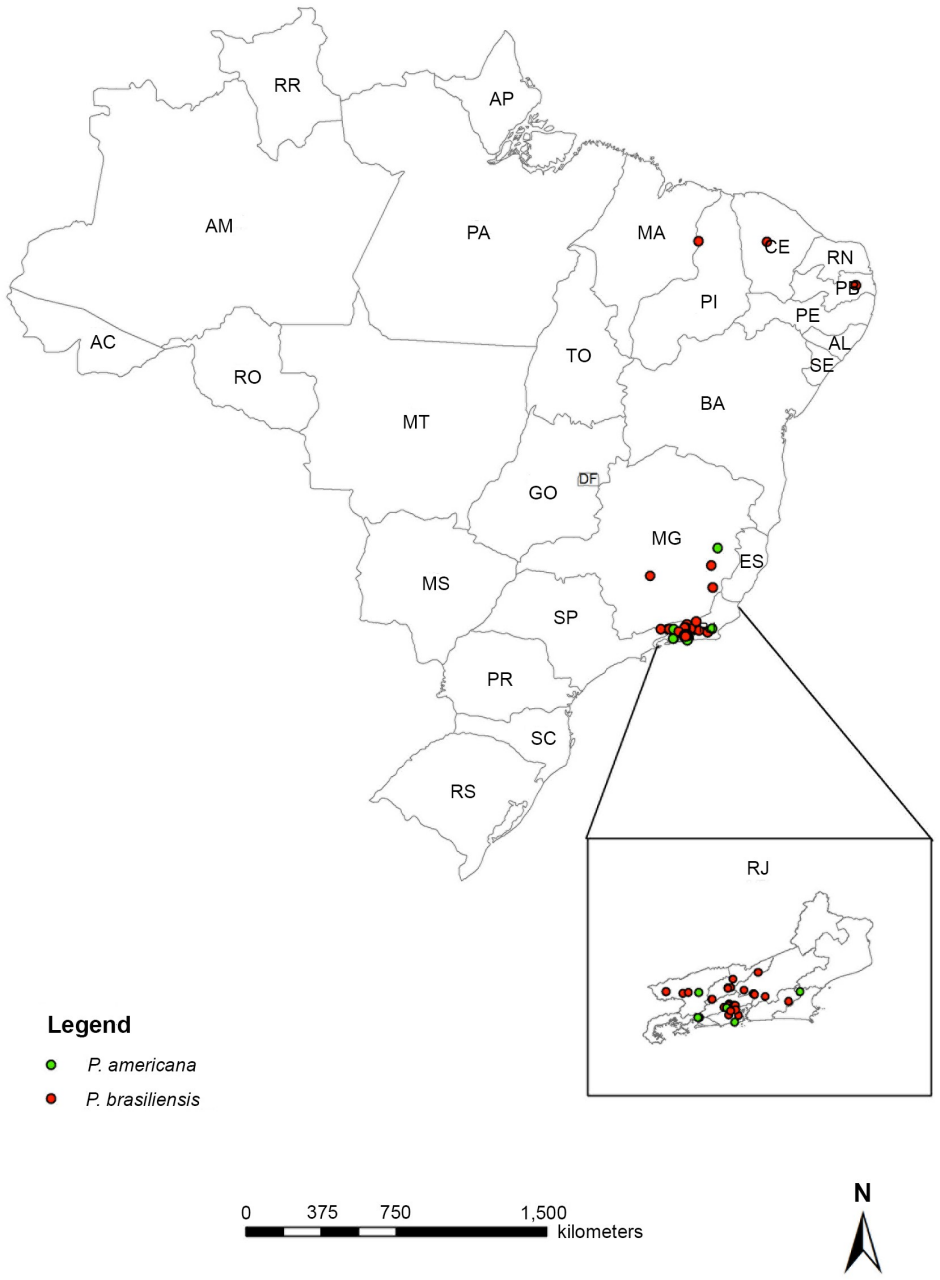
Map showing the geographic distribution of the strains analyzed in this study in the Brazilian territory according to the species molecularly identified. The states with identified strains are Piauí (PI), Ceará (CE), Paraíba (PB), Minas Gerais (MG), and Rio de Janeiro (RJ). This last state is maximized in the right inferior box. Each dot represents the probable origin of one strain. Red dots: *Paracoccidioides brasiliensis*, green dots: *Paracoccidioides americana*. Base map data from Brazilian Institute of Geography and Statistics (IBGE) and Secretary of State for Regional Development, Supply and Fisheries. Produced by Geoprocessing Laboratory/LIS/ICICT/FIOCRUZ.

To the best of our knowledge, this is the largest assessment of *P*. *americana* ever reported and the main *P*. *brasiliensis* report of cases with molecular identification and description of the respective medical features of PCM. The geographic origin, that is, the probable place of infection, of *P*. *brasiliensis* strains included the following Brazilian states: Rio de Janeiro (34 cases), Minas Gerais (5 cases), Paraíba, Ceará, and Piauí (1 case, each). Cases of PCM due to *P*. *americana* were from Rio de Janeiro (5 cases) and Minas Gerais (1 case). Fig 1 shows the geographic distribution of the *Paracoccidioides* spp. strains related to the probable source of infection of the cases here studied.

**Fig 2:**
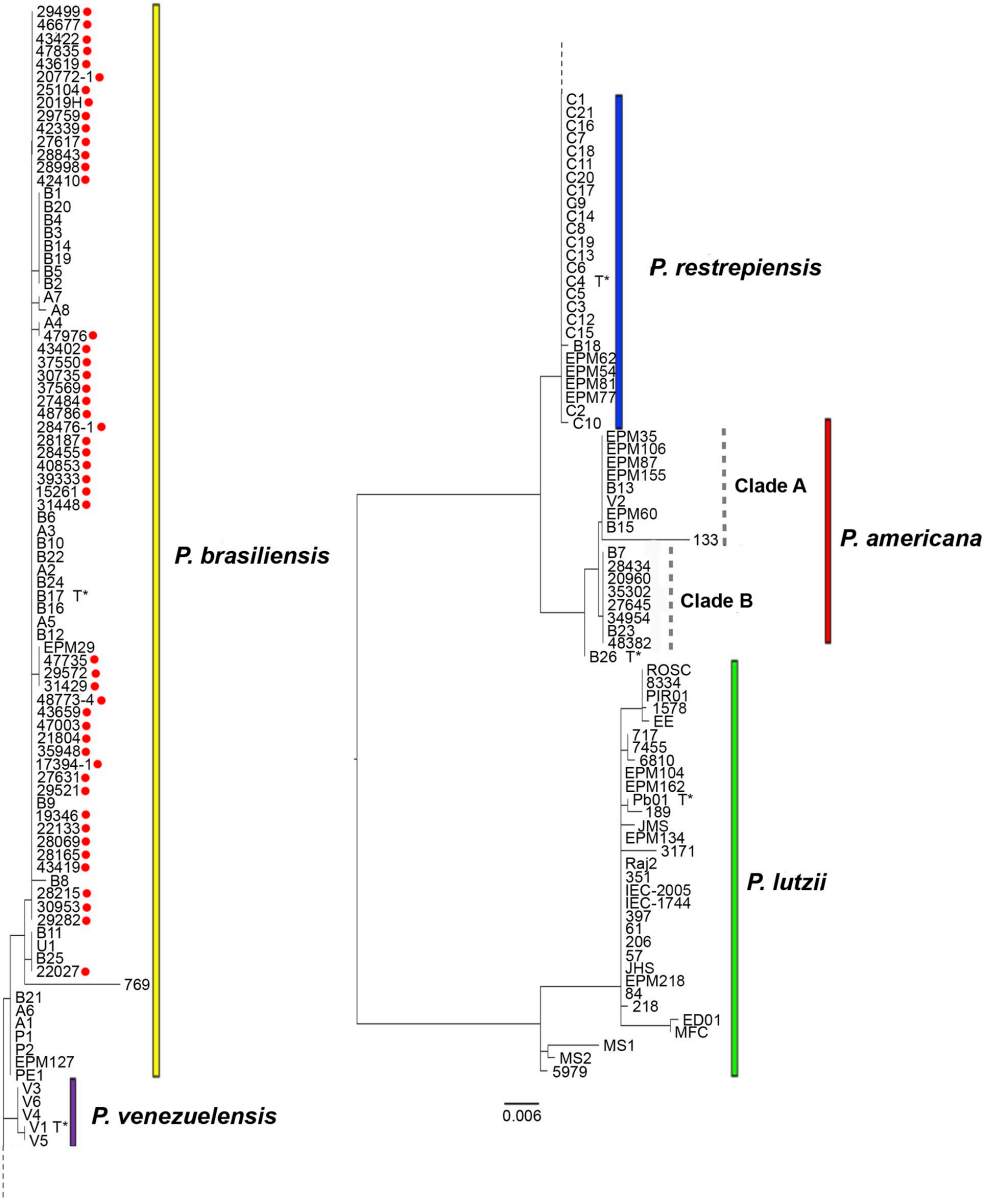
Phylogenetic tree including the strains identified in this study and reference strains. The relationships of *arf* and *gp43* genes among the 54 clinical isolates included in this study (marked with red dots) and 110 strains previously identified are represented. Type strains for each species are marked with T*. The phylogenetic tree was estimated in the FigTree version 1.4 software. Colors in bars represent the *Paracoccidioides* species described so far. Yellow: *P*. *brasiliensis*, red: *P*. *americana*, blue: *P*. *restrepiensis*, purple: *P*. *venezuelensis*, green: *P*. *lutzii*. Clades A and B of *P*. *americana* are also represented (gray dotted vertical bars).

The strains we define as belonging to the *P*. *brasiliensis* clade are genetically undifferentiated from the ones recovered previously from São Paulo state, Brazil. Also, the strains identified as *P*. *americana*, recovered from patients infected at the Rio de Janeiro state (n = 5) or at Minas Gerais (n = 1), clustered in the same clade (clade B) with two other strains previously identified from São Paulo (B7 and B23). This clade is distinct from clade A, which contains strains mainly from São Paulo suggesting that cryptic genotypes within this species may exist within *P*. *americana*. Fig 2 represents the phylogenetic analysis of the strains identified in this study.

**Fig 3:**
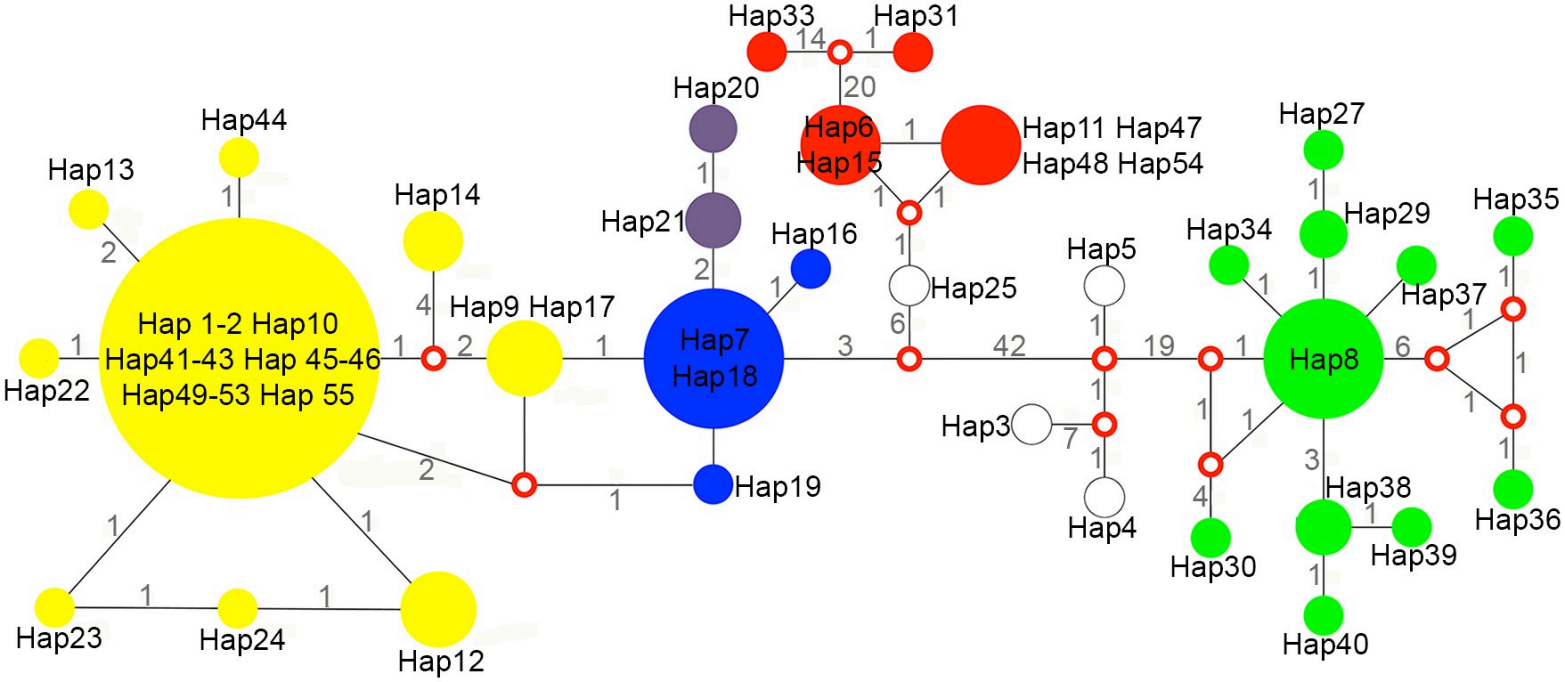
Haplotype network including the 54 strains identified in this study and 110 reference strains. Median-joining haplotype network of *Paracoccidioides* isolates based on concatenated *arf* and *gp43* partial gene sequences. The circumference sizes are proportional to the frequency of haplotypes. Red open dots (median vectors) represent unsampled or extinct haplotypes in the studied population. Numbers between circles represent the amount of mutational steps. All newly described *Paracoccidioides* species formed segregated clusters in the network: *P*. *brasiliensis* (yellow), *P*. *americana* (red), *P*. *restrepiensis* (blue), *P*. *venezuelensis* (purple), and *P*. *lutzii* (green).

The species identification based on haplotype network and phylogenetic analysis were similar (Fig 3). In brief, all *P*. *americana* strains from Rio de Janeiro and Minas Gerais clustered within a single haplotype complex (Hap11, Hap47, Hap48 and Hap54 were collapsed) within *P*. *americana* (Fig 2). The grouping of the strains B7 and B23 were also noted in the network analysis since those are also placed within the haplotype containing the newly *P*. *americana* defined strains. The *P*. *americana* type-strain isolated at São Paulo (Pb3) grouped with a different haplotype. The majority of *P*. *brasiliensis* (n = 46, 95.8%) strains genotyped were placed within the major haplotype complex of *P*. *brasiliensis* (Hap1-2, Hap10, Hap41-43, Hap45-46, Hap49-53 and Hap55 were collapsed–Fig 2). This included patients likely infected in Rio de Janeiro state as well as those that were likely infected in Paraíba and Ceará states. This large haplotype group also harbors the majority of clinical strains from São Paulo and Minas Gerais [12, 14, 40, 41], indicating that this is the predominant *P*. *brasiliensis* haplotype in Brazil. By analyzing the two loci, *arf* and *gp43*, we did not differentiate the *P*. *brasiliensis* S1a and S1b cryptic genotypes as reported using whole genome typing [16].

One *P*. *brasiliensis* strain in this study (47735) formed an exclusive haplotype. This strain was isolated from a patient whose probable place of infection was the Rio de Janeiro state (Duque de Caxias municipality), although the major clinical presentation of this case, portal hypertension, was very similar to another case caused by a strain belonging to the large *P*. *brasiliensis* haplotype [23].

A different *P*. *brasiliensis* strain in our study that did not cluster in this large haplotype (22027) was isolated from a patient living in Rio de Janeiro at the time of diagnosis, but who probably acquired the infection in the Brazilian Northeast state of Piauí, known for rare occurrences of autochthonous cases of PCM [1]. This patient presented the chronic form and did not report risk activities associated with PCM in the state of Rio de Janeiro or in any other state. However, in childhood, he lived in rural conditions for several years in his place of birth (Teresina, the capital of Piauí state). This strain was genetically similar to the following reference strains: B11 (an armadillo isolate, origin from Pará), B25 (isolated from a chronic human case of São Paulo) and U1 (isolated from penguin feces, Antarctica) and were also placed into a single branch on the phylogenetic analysis [12]. Considering the absence of PCM cases acquired in the northeastern semi-arid region [1], we hypothesized that this patient may have traveled in transition areas of Brazilian savanna and Amazon biomes, in the northern region of Brazil, near his childhood home.

The Southeastern region of Brazil is dominated by two main biomes: The Brazilian neotropical savanna and the Atlantic rainforest, with no clear geographic barriers that impair fungal migration through these areas. However, these two highland biomes are characterized by different climatic conditions, soil types, and a diverse floral and faunal composition. The three main highland Brazilian areas are: (a) The Atlantic Plateau, extending all along the eastern coast of Brazil, (b) Southern Plateau, advancing inland towards the southern and southern-central areas, and (c) the Central Plateau that is placed in the central regions of Brazil, which is mostly covered by the Brazilian savanna vegetation. For instance, past studies revealed that within the endemic area of Botucatu, São Paulo state, both humans and armadillos can be infected by both *P*. *brasiliensis* and *P*. *americana* [38]. A single armadillo captured in the surrounding areas of Botucatu, carried both *P*. *brasiliensis* and *P*. *americana* isolates suggesting that those species are likely sympatric.

Both *P*. *brasiliensis* and *P*. *americana* were found in this study to be the causative agents of PCM in patients described in a recent outbreak of acute PCM occurring after the construction of a highway in the Rio de Janeiro metropolitan area [5] which reinforces that those species occupy the same geographical areas. In other to achieve a better understanding of the influence of ecological niches on speciation, more samples are needed, and a greater number of alleles need to be assessed, which may be facilitated by whole genome sequencing.

### Socio-demographic aspects

Regarding the sociodemographic and epidemiological aspects, the mean age was 38 years of age (95% CI 34–42) for the cases due to *P*. *brasiliensis*, and 43.5 years (CI 95% 33–54) among those due to *P*. *americana*. This is in accordance with several studies reporting that PCM affects mostly working adults [1, 7, 18]. There were no statistical differences between the variables analyzed related to the identified *Paracoccidioides* species. The main variables studied are detailed in Table 1.

**Table 1:** Sociodemographic and epidemiological data of patients with paracoccidioidomycosis according to the species identified in this study.

Variable analyzed	*P*. *brasiliensis*		*P*. *americana*		*p* value^b^
	n	% (95%CI^a^)	n	%(95%CI)	
Sex (male)	36	88 (78–98)	4	67 (29–100)	0.2136
Rio de Janeiro state origin	29	71 (55–84)	5	83 (54–100)	1.0000
Minas Gerais origin	4	10 (1–20)	1	17 (0–46)	0.5115
Risk activities present	20	49 (33–64)	4	67 (29–100)	0.6662
Alcohol use	22	54 (37–69)	5	83 (54–100)	0.2205
Tobacco use	29	71 (55–84)	5	83 (54–100)	1.0000

^a^ CI–confidence interval

^*b*^ *p* value based on Fisher’s exact test

### Clinical aspects

No statistically significant differences were detected regarding clinical aspects and the species identified. Table 2 summarizes the main clinical findings of the cases studied according to the involved fungal strain.

**Table 2:** Clinical aspects of patients with paracoccidioidomycosis according to the species identified in this study.

Variable analyzed	*P*. *brasiliensis* (n = 41)		*P*. *americana* (n = 6)		*p* value*
	n	% (95% CI)	n	% (95% CI)	
**Clinical form**					
Chronic	23	56 (41–71)	5	83 (54–100)	0.3783
Acute/subacute	16	39 (24–54)	1	17 (0–46)	0.3958
Mixed	2	5 (0–11)	0	0	1.0000
**Severity**					
Mild	7	17 (6–29)	1	17 (0–46)	1.0000
Moderate	18	44 (29–59)	4	67 (29–100)	0.3980
Severe	16	39 (24–54)	1	17 (0–46)	0.3958
**Organs affected**					
UAWT	26	63 (49–78)	5	83 (54–100)	0.6484
Larynx	7	17 (6–29)	2	33 (0–71)	0.3222
Lungs	23	56 (41–71)	5	83 (54–100)	0.3783
Lymph nodes	21	51 (36–67)	2	33 (0–71)	0.6662
Skin	16	39 (24–54)	0	0	0.0819
Liver/spleen	10	24 (11–38)	0	0	0.3173
Adrenal	6	14 (6–29)	3	50 (12–88)	0.0747
Bone marrow	3	7 (0–15)	0	0	1.0000
CNS	2	5 (0–11)	0	0	1.0000
Bone	2	5 (0–11)	0	0	1.0000
**Coinfections**					
Tuberculosis	3	7 (0–15)	0	0	1.0000
HIV/AIDS	5	12 (4–26)	0	0	1.0000
Viral hepatitis	2	5 (0–11)	1	17 (0–46)	0.3426
Intestinal worms	6	14 (6–29)	2	33 (0–71)	0.2672

CI–confidence interval; UAWT–upper airway tract; CNS–central nervous system

* *p* value based on Fisher’s exact test

In Rio de Janeiro, the proportion of the acute form of PCM is historically reported as 3–10% of all PCM cases [5, 8, 18]. The high proportion of acute/subacute juvenile clinical forms as well as severe cases and HIV-AIDS coinfected patients in the present study are possibly related to the higher fungal burden of these cases that facilitates fungal isolation in culture, which was an inclusion criterion of this study. Both *Paracoccidioides* species identified in this study were involved in acute/subacute PCM cases. To the best of our knowledge, this is the first formal description of acute/subacute PCM due to *P*. *americana*. There is a previous report of a PCM case caused by *P*. *brasiliensis* PS2 (now *P*. *americana*) in a young male patient, however other clinical aspects of this infection were not reported, which impairs the correct classification of the clinical form in this case [14]. Due to the age of the patient, it is thought that this was an acute/subacute PCM case. Taken together, these two reports support the proposition that *P*. *americana* can also cause acute PCM.

On average, adrenal involvement related to PCM is reported in 56% of autopsied cases [7, 26]. Also, it is worth mentioning that the high proportion of adrenal impairment in the small group of cases due to *P*. *americana* suggests a possible adrenal tropism of this species. It is well described that *P*. *brasiliensis* [8, 21, 23, 25] and *P*. *lutzii* [45] can be related to severe PCM cases. Adrenal involvement can bring severe sequelae, which also could associate *P*. *americana* as well as *P*. *brasiliensis* and *P*. *lutzii* with severe PCM cases.

### Therapeutic aspects

Table 3 shows the main characteristics of treatment and the respective total time of treatment according to each species. One *P*. *brasiliensis* infected patient did not receive antifungal treatment because she never returned to our institution after fungal isolation and diagnosis. The treatment times are in accordance with other studies on PCM therapy [7, 26]. A previous study suggested that *P*. *brasiliensis* was less responsive than *P*. *lutzii* to SMZ/TMP using an *in vitro* susceptibility test [46]. In the present study the six *P*. *brasiliensis* infected patients had a good response to this drug. It is not possible to know the exact species within the former *P*. *brasiliensis* complex (S1, PS2, PS3, or PS4) studied by Hahn and collaborators, therefore this therapeutic response to SMZ/TMP needs to be further explored under the light of the new *Paracoccidioides* species. The high frequency of drug combination in this study reflects the severity and complexity of the cases since drug association is known to be a good strategy for critical and neurological cases [8].

**Table 3:** Therapeutic aspects of patients with paracoccidioidomycosis according to the species identified in this study.

Drug prescribed	*P*. *brasiliensis* (n = 41)		*P*. *americana* (n = 6)		*p* value^a^
	n	mean time (months)	n	mean time (months)	
ITZ	12	15.7	1	15	NC^b^
SMZ/TMP	6	19.3	2	27.5	0.4841
ITZ and SMZ/TMP^c^	10	20.7	3	18.6	0.8304
Others^d^	12	44.2	0	-	NC

ITZ–itraconazole; SMZ/TMP–sulfamethoxazole-trimethoprim

^*a*^ *p* value based on Student’s t test

^b^ NC–Not calculated due to small sample size in *P*. *americana* group.

^c^ Sequentially prescribed due to adverse effects or refractoriness

^d^ Drug combinations and amphotericin B

### Serological aspects

**Fig 4:**
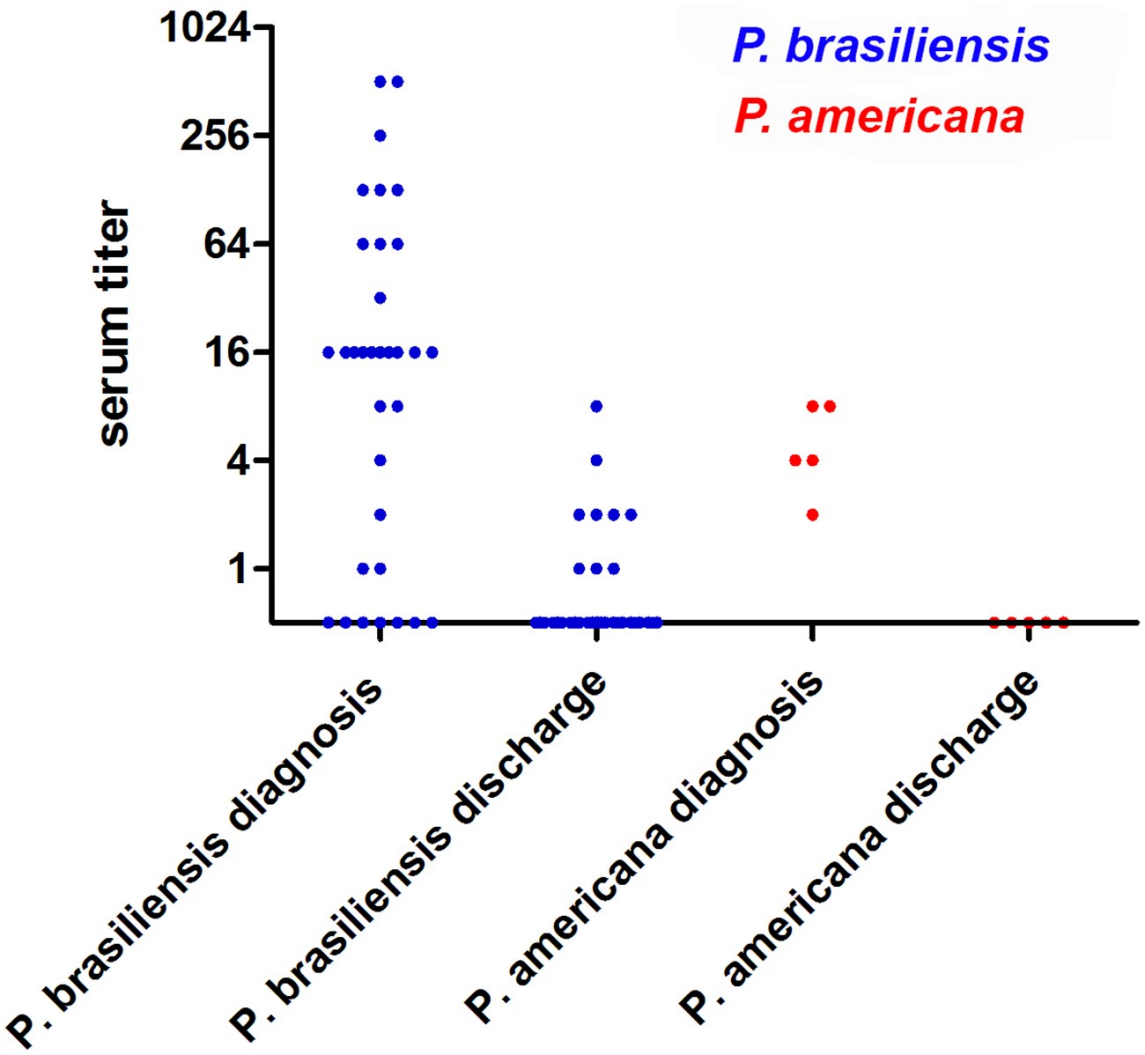
Immunodiffusion reactivity in serological samples from 38 patients of this study. Serum antibody titers from patients infected with *P*. *brasiliensis* (blue dots) and *P*. *americana* (red dots) are informed.

Immunodiffusion (ID) was positive at the time of diagnosis in 80% of PCM cases caused by *P*. *brasiliensis* (95% CI 68–92) and in 100% of PCM cases caused by *P*. *americana* (95% CI 54–100), showing no significant differences in sensitivity of ID regarding *Paracoccidioides* species. These results highlight the efficacy of ID in *P*. *americana* infected patients using antigens derived from other phylogenetic species. Fig 4 shows the reactivity of the test according to the etiologic agent in 38 patients who had paired titers for comparison. The first (at admission) and last (at discharge) available ID tests were considered, regardless of the outcome. For both groups, serum antibody titers at discharge were lower than at admission (*p* values of <0.0001 and 0.0173 for *P*. *brasiliensis* and *P*. *americana*-infected patients, respectively).

Eight cases caused by *P*. *brasiliensis* did not react to ID, although these results were expected for three cases due to HIV/aids coinfection [7]. Since aids can impair antibody production, reducing ID sensitivity, this parameter was also calculated excluding the 5 patients living with HIV/aids. In this scenario, ID of PCM caused by *P*. *brasiliensis* presented a sensitivity of 86% (95% CI 71–95). Two previous reports regarding results of serological reactivity associated with the molecular species responsible for PCM reveal differences. While the former presents a case, whose fungal agent identified by serologic tools was *P*. *lutzii* [47], the latter reports a case due to *P*. *brasiliensis* molecularly identified in which serology reacted only against *P*. *lutzii* antigens [48]. More studies are necessary to clarify these findings. However, our findings reinforce that molecular techniques are likely most appropriate.

### Prognostic aspects

No differences between species were found regarding prognostic aspects (Table 4). Since its description, *P*. *lutzii* has been implicated in poor prognosis of PCM [15, 45]. *P*. *brasiliensis* was responsible for many complications in the patients included in this study, severe cases including acute lymph abdominal forms and a fatal septic shock similar to a previous case report of PCM associated with *P*. *lutzii* [25, 45].

**Table 4:** Prognostic aspects of patients with paracoccidioidomycosis according to the species identified in this study.

Variable analyzed	*P*. *brasiliensis* (n = 41)		*P*. *americana* (n = 6)		*p* value^b^
	n	% (95% CI^a^)	n	% (95% CI)	
**Outcome**					1.0000
Cure	26	63 (49–78)	5	80 (45–100)	
Death due to PCM	4	10 (3–23)	0	0	
**Complications**					
Dysphonia	7	17 (6–29)	2	33 (0–71)	0.3222
Low adrenal reserve	5	16 (3–28)	3	40 (0–83)	0.0812
Cholestasis	4	10 (1–19)	0	0	1.0000
Palatal perforation	3	7 (0–15)	0	0	1.0000
Tracheostomy	2	5 (0–11)	0	0	1.0000
Microstomy	2	5 (0–11)	0	0	1.0000
Portal hypertension	2	5 (0–11)	0	0	1.0000

^a^ CI–confidence interval

^*b*^ *p* value based on Fisher’s exact test

*P*. *americana* was also associated to dysphonia and low adrenal reserve. In Botucatu, a municipality of São Paulo state, dysphonia was reported as a frequent PCM complication associated with laryngeal involvement [49]. It is not possible to infer the *Paracoccidioides* species associated with those cases, but Botucatu is located in the Brazilian southeast, an area of *P*. *americana* occurrence [7, 22]. Another study conducted in São Paulo reported up to 44% significant hypoadrenalism in patients with PCM [50], a frequency similar to that found in the *P*. *americana* patients of this study. In Rio de Janeiro, low adrenal reserve was observed in approximately 13% of patients with acute PCM [8], a frequency similar to that observed in the *P*. *brasiliensis* infected patients herein described, which can be explained by the predomination of *P*. *brasiliensis* in the patients included in the present work.

### Ecological factors that underlies *P*. *brasiliensis* and *P*. *americana* distribution

Microbial communities are made up of distinct genetic entities and defining species boundaries and range in fungal pathogens is essential for molecular epidemiology studies. Not only for its clinical relevance in Latin America, *Paracoccidioides* may offer an interesting model of complex genetic microbial entities.

**Fig 5:**
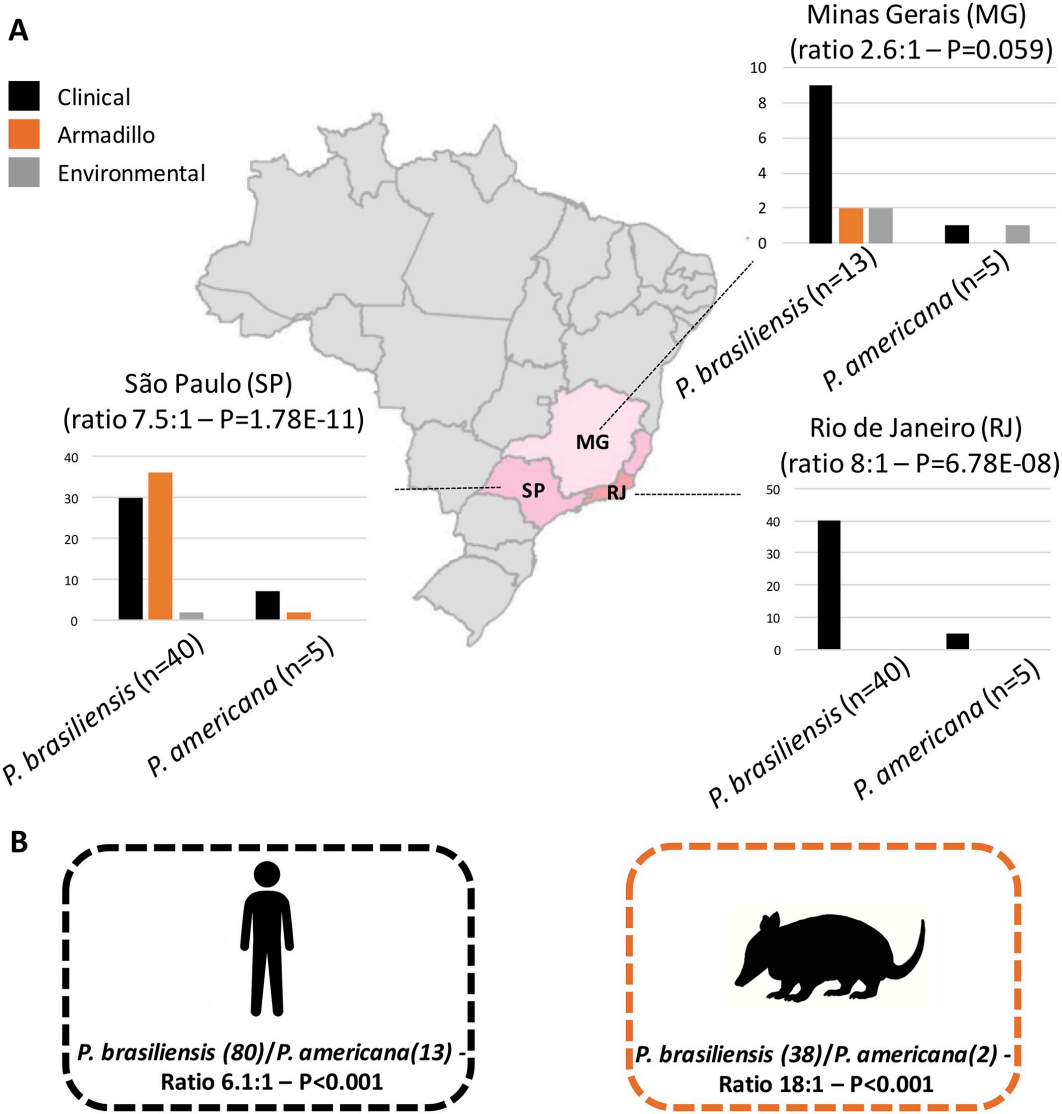
*Paracoccidioides brasiliensis/Paracoccidioides americana* ratio among environmental, human, and armadillo strains. (A) ratios of three states from the Southeastern Brazilian region, where the study was conducted: Minas Gerais (MG), Rio de Janeiro (RJ), and São Paulo (SP). Graphs represent the number of identified strains from clinical (black bars), armadillos (orange bars), and environmental (gray bars) origins. (B) global ratios and number of human and armadillo *P*. *brasiliensis* and *P*. *americana* strains. Base map data from Brazilian Institute of Geography and Statistics (IBGE).

Strikingly, by analyzing our study and retrospective reports that used molecular techniques to differentiate the *Paracoccidioides* species, we observed that the ratio of *P*. *brasiliensis*/*P*. *americana* distribution is uneven considering either São Paulo, Rio de Janeiro, or Minas Gerais as distinct states as well taking account human or armadillo populations suggesting that those species may occupy different niches (Fig 5).

According to our data, *P*. *brasiliensis* is more prevalent than *P*. *americana* in the three analyzed states of Brazil ranging from 2.6:1 in Minas Gerais, 7.5:1 in São Paulo and 8:1 in Rio de Janeiro (Fig 5). We compared those proportion via N-1 Chi-square test and observed that this uneven species distribution is statistically significant in São Paulo (Difference—76.60%, 95% CI—42.7234 to 87.6818, χ^2^–28.088, DF 1, P < 0.0001) and Rio de Janeiro (Difference—78.80%, 95% CI—33.2949 to 90.0857, χ^2^–17.656, DF 1, *p* < 0.0001) but not in Minas Gerais (Difference—44.49%, 95% CI—-4.4054 to 71.7512, χ^2^–2.81, DF 1, *p* = 0.0937). We also used Chi-square tests in order look for deviations from the 1:1 ratio for the *P*. *brasiliensis*/*P*. *americana* species distribution. The uneven distribution was found significantly in São Paulo (*p* < 0.0001) and Rio de Janeiro (*p* < 0.0001) but not in Minas Gerais, where a tendency was noted (*p* = 0.059) (Fig 5). We also observed a skewed species distribution by considering either humans (6.1:1 –*p* < 0.0001) or armadillos (18:1—*p* < 0.0001), suggesting that in both mammal hosts this pattern is observed.

Relevant differences in phenotypes have been already reported in the literature: (i) Strains from *P*. *brasiliensis* species produce more conidia compared to *P*. *americana* (ii) *P*. *americana* produces atypical yeast morphology at 37°C compared to *P*. *brasiliensis* species [14, 17]. Variable loci may produce phenotypic plasticity in natural populations mainly due genetic drift. Under neutral selection, the genetic diversity inherited by a given population is dependent on the population size and mutation rate. In fungi, especially in dimorphic fungi, mutation rates and/or cell subdivision estimates are scarce making it difficult to determine effective population size. In sympatric species, such as *P*. *brasiliensis* and *P*. *americana*, understanding evolutionary aspects that may explain genetic plasticity is facilitated by comparing genomes of sister species that diverge in life history or ecology in the same geographical area. Recent population genomic studies revealed these closely related species have similar low nucleotide diversity indexes (*P*. *brasiliensis*—π = 0.00053 and *P*. *americana—*π = 0.00066) and genome-wide calculation of Tajima’s D did not deviate from the null hypothesis suggesting neutrality. By combining this nucleotide diversity and phylogenomic measurements the authors suggested that *P*. *americana* is a more ancient species compared to *P*. *brasiliensis* [16] and this may impact the population size and fitness of this species. These observations, coupled with a skewed species distribution, suggest that *P*. *brasiliensis* may have more efficient mechanisms to survive in the environment and to infect mammals compared to *P*. *americana*. Moreover, those species may compete for the same host and thus it may explain the differences on population size of both species.

### Conclusions

In conclusion, 54 clinical strains were newly genotyped through sequencing of both *arf* and *gp43* loci, reinforcing that both *P*. *brasiliensis* and *P*. *americana* are endemic species in Rio de Janeiro. The majority of *P*. *brasiliensis* strains from Rio de Janeiro state clustered within *P*. *brasiliensis* with no genetic differentiation from those from São Paulo. However, *P*. *americana* recovered from Rio de Janeiro formed a new cluster apart from that previously described containing strains from São Paulo, Minas Gerais (Brazil), and Venezuela suggesting that the genetics of this species is more complex than previously thought. For the first time, clinical and molecular aspects of PCM in the endemic area of Rio de Janeiro are described. In this geographical region, *P*. *brasiliensis* was responsible for severe lymph abdominal forms including massive splenomegaly, portal hypertension and fatal septic shock. *P*. *americana* appears to have adrenal tropism, presented 100% reactivity to immunodiffusion, even when tested against antigens from other species, and caused acute forms, along with *P*. *brasiliensis*. No statistically significant associations were found between the two species analyzed and clinical aspects. Comparative analysis considering retrospective genotyped cases of human and armadillo infections suggests that those two species have different population sizes and may compete from the same host.

This study was performed with regional-origin patients and present limitations with regards to the number of cases analyzed, especially in those caused by *P*. *americana*, as well as the representativeness of the species of the genus *Paracoccidioides*. However, the prevalence of *P*. *americana* in the Brazilian Services is unknown. Therefore, this number could not be so small, and the publication of these data may launch the knowledge of this species distribution and its clinical aspects. In addition, aspects such as the strain virulence, the inhaled fungal burden, the genetic and immunological susceptibility of the host as well as the diagnostic delay and other social determinants of health inequality deserve future studies to address their conjunct role in the spectrum of clinical presentations and in the severity of PCM.

Future multicenter studies including a higher number of fungal strains including all species and their corresponding clinical data are required to fully understand this severe neglected systemic mycosis, so relevant to public health.

## Supporting information

S1 Table Accession numbers of the *Paracoccidioides sequences analyzed in* this study. (XLSX)
